# In Situ Measurement Methods for the CO_2_-Induced Gelation of Biopolymer Systems

**DOI:** 10.3390/gels6030028

**Published:** 2020-09-09

**Authors:** Imke Preibisch, Lena-Marie Ränger, Pavel Gurikov, Irina Smirnova

**Affiliations:** 1 Institute of Thermal Separation Processes, Hamburg University of Technology, 21073 Hamburg, Germany; irina.smirnova@tuhh.de; 2 Institute of Chemical Engineering, Laboratory of Thermal Process Engineering, Ulm University, 89081 Ulm, Germany; lena.raenger@uni-ulm.de; 3 Laboratory for Development and Modelling of Novel Nanoporous Materials, Hamburg University of Technology, 21073 Hamburg, Germany; pavel.gurikov@tuhh.de

**Keywords:** CO_2_-induced gelation, hydrogel, biopolymer, amidated pectin, high pressure

## Abstract

This work presents two novel methods to investigate in situ the carbon dioxide (CO_2_)-induced gelation of biopolymer-based solutions. The CO_2_-induced gelation is performed in a viewing cell at room temperature under CO_2_ pressure (20 to 60 bar), whereby calcium precursors are used as cross-linkers. The novel methods allow the in situ optical observation and evaluation of the gelation process via the change in turbidity due to dissolution of dispersed calcium carbonate (CaCO_3_) particles and in situ pH measurements. The combination of both methods enables the determination of the gelation direction, gelation rate, and the pH value in spatial and temporal resolution. The optical gelation front and pH front both propagate equally from top to bottom through the sample solutions, indicating a direct link between a decrease in the pH value and the dissolution of the CaCO_3_ particles. Close-to-vertical movement of both gelation front and pH front suggests almost one dimensional diffusion of CO_2_ from the contact surface (gel–CO_2_) to the bottom of the sample. The gelation rate increases with the increase in CO_2_ pressure. However, the increase in solution viscosity and the formation of a gel layer result in a strong decrease in the gelation rate due to a hindrance of CO_2_ diffusion. Released carbonate ions from CaCO_3_ dissolution directly influence the reaction equilibrium between CO_2_ and water and therefore the change in pH value of the solution. Increasing the CaCO_3_ concentrations up to the solubility results in lower gelation rates.

## 1. Introduction

Aerogels are solid materials with high specific surface area and porosity. Recently, they become more and more promising for the applications in food and pharmaceutical area. Biopolymers in particular are receiving considerable attention as precursors for the aerogel production due to unique properties such as edibility and biodegradability (besides the ability to form a gel, which is common for various natural food components). The general steps of aerogel production from biopolymers can be summarized as follows: (1) dissolution of the biopolymer, (2) gelation of the resulting solution, (3) solvent exchange, and (4) supercritical drying with carbon dioxide (CO_2_) [1]. The first step of biopolymer dissolution mainly depends on the physical properties of a selected biopolymer. However, the gelation step, which has a decisive impact on the formed structure and therefore the properties of obtained aerogels, is often similar for different biopolymer classes. Depending on specific properties of biopolymers, various gelation methods have been developed such as thermal, ionotropic, or acid-induced gelation. One promising method suggested for the gelation of biopolymer solutions is the so-called CO_2_-induced gelation [2]. Here, pressurized CO_2_ is applied to the biopolymer solution, causing its gelation. It has been applied to different biopolymers including alginate [2,3,4], amidated pectin [1], silk fibroin [5,6,7], α-elastin [8,9], chitosan [10], whey protein isolate [11], potato starch [12], myosin [13], collagen [14], and hybrids of different biopolymers [3,15]. It has been shown that CO_2_-induced gelation has several advantageous compared to common gelation methods for biopolymers. CO_2_-induced gelation enables faster gelation [5,6] resulting in more homogeneous and stable hydrogels [2,5,6,9,11] with higher porosities [5,6], enhanced pore connectivity [9], and increased specific surface area [15] when compared to conventionally gelled counterparts. Supercritically dried pectin gels (after solvent exchange) have been suggested for a range of applications such as biocompatible thermal insulation [16], for drug delivery [17], and food packaging [18].

Depending on the gelation mechanism of a particular biopolymer, a combination of CO_2_-induced gelation and ionotropic gelation with additional initially non-dissolved cross-linkers like calcium carbonate (CaCO_3_) particles can be employed [1,2].

**Figure 1:**
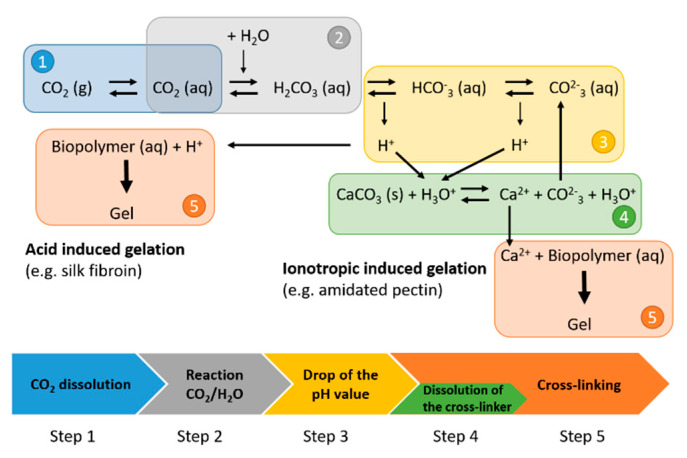
Scheme of the gelation process of CO_2_-induced gelation of biopolymer solutions with possibly cross-linker calcium carbonate.

The overall mechanism of the CO_2_-induced gelation can be divided in five steps (Figure 1):

1. Dissolution of CO_2_ in the aqueous biopolymer solution;
2. Reaction of CO_2_ with water and formation of carbonic acid;
3. Dissociation of the carbonic acid resulting in a pH drop;
4. Dissolution of cross-linkers (e.g., CaCO_3_) due to increased solubility at a reduced pH value;
5. Cross-linking of biopolymer chains, which is either induced by reduction of the pH value (step 3) and therefore interaction of biopolymer molecules or by interaction of biopolymer molecules with released cations (step 4).

CO_2_ dissolution in and reaction with water (steps 1–3, Figure 1) and the dissolution of CaCO_3_ (step 4, Figure 1) in systems without biopolymers have been studied widely in the literature [19,20,21,22,23,24]. It was shown that additional components like inorganic salts have an impact on the solubility of CO_2_ and CaCO_3_ in aqueous solutions [19,22,23,24]. Regarding the reaction equilibrium of the reaction system of CO_2_ and water, it was shown that at pH 3.5–7, the main carbonate containing species is the bicarbonate ion [19]. The solubility of CaCO_3_ increases with decreasing pH value [25,26] and increasing pressure of CO_2_ [27]. Furthermore, although released carbonate ions from CaCO_3_ dissolution directly influence the equilibria in the reacting water/CO_2_ system and thus affect the pH value [28], bicarbonate is the main carbonate containing species in the ternary solution at pH 2.5–7 [19].

Each step of the CO_2_-induced gelation process of biopolymer solutions is most likely impacted by the biopolymer directly due to buffering effects [5] and by progressive changes of physical properties of the reacting solution such as density and viscosity. Furthermore, most probably, each step has in turn a strong impact on the overall gelation process and possibly on the aerogel properties. Nevertheless, although the CO_2_-induced gelation is a promising approach, its detailed mechanism as well as the impact of the pressurized CO_2_ still remain not elucidated.

Only a few works investigate the mechanism of the CO_2_-induced gelation of biopolymer solutions. Floren et al. [5] studied the gelation of silk fibroin solutions in CO_2_ atmosphere at pressures from 5 to 150 bar at 40 °C without any additional cross-linking agents. A change in the visual appearance of the protein solution from a transparent solution to an opaque gel in CO_2_ atmosphere was observed. This change in appearance was homogeneous over the entire sample and was used as an indicator for the ongoing gelation process. A homogeneous drop of the pH value throughout the aqueous silk fibroin solution and therefore gelation due to protein precipitation was assumed. The determination of the gelation progress was performed ex situ after depressurization of the system: the solution was defined as gelled if the sample does not flow from the inverted beaker. Hence, the ex situ gelation times are likely to be biased by non-isobaric conditions and a certain duration of the depressurization. Shorter gelation time with increasing CO_2_ pressure was observed. Furthermore, the impact of high pressure itself on the gelation time was proven by comparing ambient pressure acid-induced gelation with high-pressure-assisted acid-induced gelation at nitrous oxide atmosphere at the same pH value. A decrease in the gelation time comparable to the effect of pressurized CO_2_ was identified, indicating a strong impact of hydrostatic pressure separated from the pH drop.

Mallepally et al. [6] compared a low pressure (2 bar) bubble through method to the stagnant high pressure method of Floren et al. [5]. An increased gelation velocity by the enhanced CO_2_ mass transfer into the solution of silk fibroin due to an increased contact surface area was demonstrated.

In situ pH measurements in combination with viscosity measurements to track the gelation process were conducted by Zubal et al. [14] and Oechsle et al. [29] in collagen and cellulose nanocrystal/imidazole solutions, respectively. In both cases, measurements were performed at atmospheric pressure either throughout evaporation of CO_2_ after depressurization or while sparging the solution with CO_2_ or nitrogen at low pressure, respectively. A clear dependence of the viscosity and therefore the gelation progress of the biopolymer solutions on the pH value was shown.

A theoretical description of the CO_2_-induced gelation of protein solutions without additional cross-linker is provided by Floren et al. [5]. The model is based on the assumption that the reaction of CO_2_ with water dominates the gelation mechanism. Especially at increased pressures, CO_2_ dissolution in water was assumed to be the limiting step of the pH reduction process. Additionally, it was assumed that the fast formation and dissociation of carbonic acid and rapid diffusion of protons result in a homogenous drop of the pH value inside the protein solution. Therefore, all protein molecules exhibit the same net electrostatic charge at all time without having an impact on the dissolution and reaction of CO_2_ and water. A theoretical calculation of the pH value inside the protein solution was performed, including the buffer capacity of the protein. Determined pH profiles over time were used to calculate the required gelation time, and it was pointing to a critical part of the mass transfer in the entire acidification process. Comparison with measured gelation times revealed that the impact of the protein concentration is captured insufficiently by the model.

Several works studied the secondary structure of the biopolymer before and after the CO_2_ treatment; see step 5 of the gelation process in Figure 1. It was shown for most proteins that the treatment with pressurized CO_2_ at temperatures below 60 °C has no specific effect on the secondary structure when compared to ambient pressure acid treatment. Neither in ex situ measurements after depressurization [5,6,8,10,11] nor in in situ measurements up to 80 bar [8], nor different gelation methods (stagnant or bubble through) [6] show an impact. However, a clear impact on the macroscopic characteristics of the gelation process such as decreased gelation temperature or decreased gelation time was observed [6,8]. Only at temperatures above 60 °C, Xu et al. [11] observed a negative effect of the high-pressure CO_2_ atmosphere resulting in the loss of structural integrity when compared to pure thermal or high-pressure nitrogen treatment.

Regarding the structural change in polysaccharides, Muljana et al. [12] showed that in CO_2_ atmosphere, the pressure has an impact on the secondary structure of potato starch and therefore the degree of gelatinization. The measurements of the secondary structure were performed after equilibration at pressures from 1 to 250 bar.

In summary, it can be said that besides information about the change in the secondary structure of biopolymers due to high-pressure CO_2_ treatment, dynamic mechanisms of the process steps of the CO_2_-induced gelation of biopolymers are not revealed. Furthermore, no in situ measurement methods to elucidate the overall gelation mechanism of the CO_2_-induced gelation of biopolymer solutions at elevated pressure have ever been presented. To understand the whole gelation process, individual process steps should be studied in situ. The aim of this work is therefore to develop two in situ methods to enable the measurement of the pH drop throughout the gelation process (step 1–3, Figure 1) and dissolution of calcium carbonate (step 4, Figure 1) individually with spatial and temporal resolution. To evaluate the developed methods and illustrate their benefit for mechanism elucidation, a model gelation system of aqueous amidated pectin solution with suspended CaCO_3_ particles as additional cross-linker is used.

## 2. Results and Discussion

In this section, the developed in situ measurement methods for the investigation of the overall gelation mechanism of the CO_2_-induced gelation of biopolymer solutions are presented. Subsequently, the possibilities of investigations of different impact factors as well as the obtained results and the findings for the overall gelation mechanism of the model system are discussed.

The dissolution of the additional cross-linker (step 1–4, Figure 1) and, therefore, the overall gelation mechanism (step 1–5) are studied with in situ optical observation in spatial and temporal resolution. The change in the pH value (step 1–3) is measured in situ with high-pressure fluorescence pH sensor in the amidated pectin solution in spatial and temporal resolution under high-pressure CO_2_.

Due to uncertainty of actual location of the reaction of CO_2_ with water and dissociation of carbonic acid, in the following, the diffusion of all carbon-containing species and protons is summarized under one umbrella term of “diffusion of CO_2_”.

### 2.1. Optical Measurement of the CaCO_3_ Dissolution and Gelation

**Figure 2:**
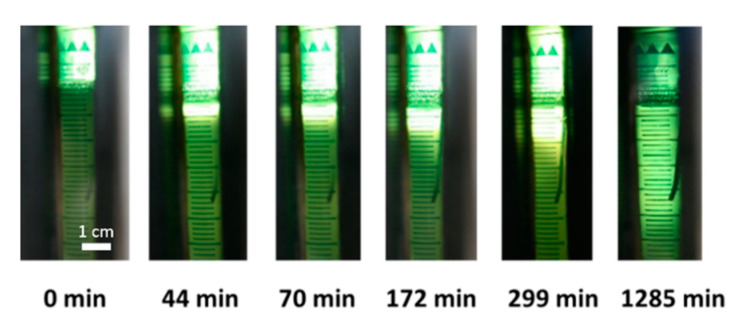
Development of the gel phase and gelation front from top to bottom over time of a 1 wt.-% amidated pectin solution with 0.18 wt.-% CaCO_3_ at 50 bar CO_2_ and room temperature.

The change in the solution turbidity during CO_2_-induced gelation of amidated pectin solutions in the presence of suspended CaCO_3_ particles is observed in situ over time as shown in Figure 2. A graduate clearance of the suspension from the top of the mold (contact area with CO_2_ gas phase) to the bottom of the mold is observed. This clearance indicates the dissolution of the CaCO_3_ particles, and therefore, the gelation of the pectin solution due to cross-linking of pectin chains with released calcium ions.

To prove the assumption that cross-linking with calcium ions (step 5, Figure 1) is much faster than dissolution (step 4), the gelation process is stopped for certain experiments by depressurization, while a gelation front could still be observed between a clear upper and a turbid lower part of the sample. After complete depressurization, the structure of the different regions is evaluated. The clear part consists of a completely gelled, self-supporting hydrogel, whereas, the turbid part is partly liquid and partly formed very weak gel, indicating that CaCO_3_ dissolution already started, but no complete gelation has yet occurred. Due to these findings and due to the fact that ionic reactions are usually very fast, it is assumed in the following work that the clearance is a direct indication of the occurred gelation. Therefore, the surface between the clear gel and the still turbid solution is defined as the gelation front in this work.

Optical observation of the gelation process over time at 50 bar reveals that gelation occurs from the top of the solution, where it is in contact with the CO_2_ gas phase, to the bottom of the solution (Figure 2). This indicates a one dimensional, vertical diffusion of CO_2_ from the top to the bottom of the polymer solution, resulting in dissolution of the CaCO_3_ particles and therefore vertical movement of the gelation front. This vertical movement of the gelation front, in comparison to homogeneous gelation, indicates that gelation is much faster than CO_2_ diffusion inside the sample. As soon as CO_2_ reached a non-gelled part of the solution, gelation occurs. Therefore, later on, CO_2_ has to diffuse through the already-formed gel at the top of the sample.

**Figure 3:**
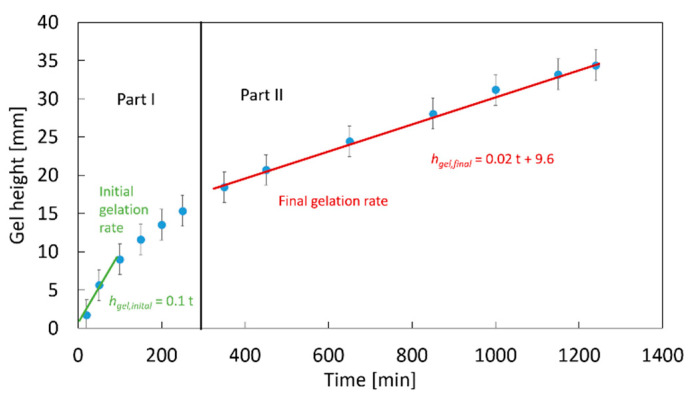
Representative case of the change in the gel height throughout gelation process at 35 bar; 1 wt.-% amidated pectin solution with 0.18 wt.-% CaCO_3_ at room temperature.

For the evaluation and quantification of the gelation process, the height of the clear gel is measured as a function of time (Figure 3), with the slope being the process gelation rate $r_{gel,period}\;$ (Equation (4)). Two regions can be identified in Figure 3: a strong increase in the gel height at the beginning of the process (part I) and, later, a nearly-linear increase in the gel height (region of constant gelation rate; part II).

**Figure 4:**
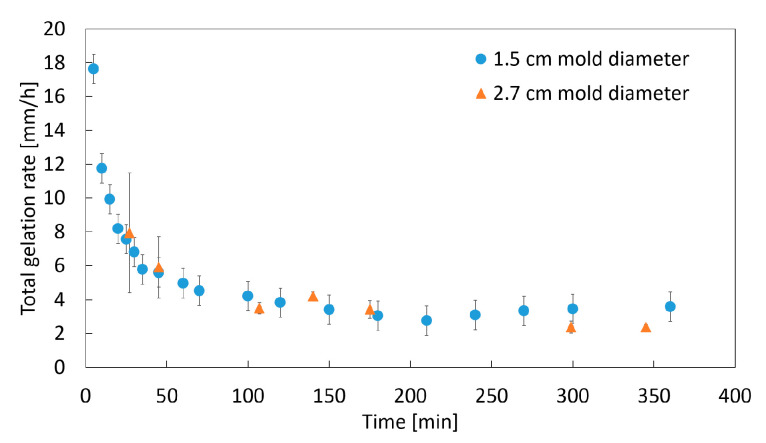
Change in the total gelation rate (Equation (3)) over time; 1 wt.-% amidated pectin with 0.18 wt.-% CaCO_3_ at 50 bar and room temperature.

The slope of linear part II is defined as the final gelation rate $r_{gel,\;final}$. The calculated total gelation rates (Equation (3)) over time are shown in Figure 4. The total gelation rate is high at the beginning of the gelation process, where the gelation front is close to the contact area with gaseous CO_2_, and it decreases strongly over time to a nearly constant gelation rate, with the propagation of the gelation front towards the bottom of the sample. The decrease in the gelation rate indicates an increasing resistance for the diffusion of CO_2_ through the already formed gel structure at the top of the solution in comparison to transport through the initially liquid phase.

#### 2.1.1. Impact of the Mold Material

To account for eventual diffusion of CO_2_ through the walls of the mold, two different types of molds are tested: polyethylene and glass molds. In the case of open polyethylene molds, after some time, additional clearance of the sample solution occurs at the sides and the bottom of the mold. To prove the possibility of diffusion through polyethylene material, additional gelation experiments are performed with closed lids for the polyethylene molds. For the closed lid polyethylene molds, after some time, a clearance of the solution can be observed, pointing to the CO_2_ diffusion through the mold material. For glass molds, no side diffusion can be observed. The significance of the effect of side diffusion depended on the material of the mold, its wall thickness and the total gelation duration (due to sample’s height).

These observations show that for the detailed investigation of the gelation mechanism, an impermeable mold material should be used to reduce overlapping of different process steps due to ongoing three dimensional “side” gelation. Furthermore, enforcing one dimensional diffusion and gelation front movement simplifies the identification of impact factors such as changing viscosity and buffering effects, as discussed later on.

#### 2.1.2. Impact of the Mold Diameter

**Figure 5:**
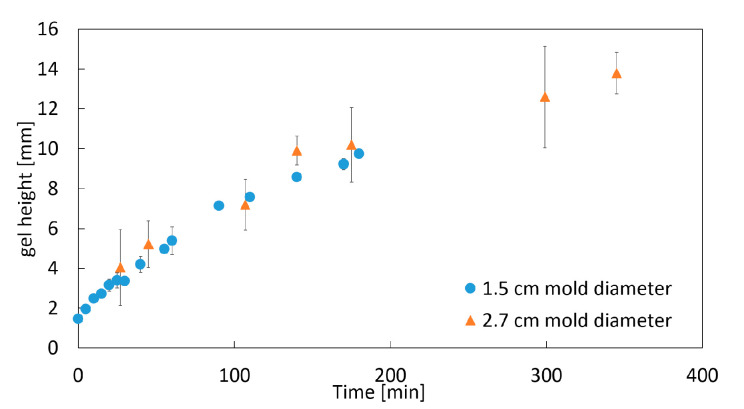
Increase in the gel height in contact with pressurized CO_2_ over time with varied mold diameters; 1 wt.-% amidated pectin solution with 0.18 wt.-% CaCO_3_ at 50 bar and room temperature.

To prove the assumption of one-dimensional diffusion of CO_2_ in the vertical direction, the mold diameter is varied to change the contact area between the sample and gaseous CO_2_. Neither the increase in the gel height nor the change in the gelation rate over time is influenced by the diameter of the mold (Figure 4 and Figure 5). These observations prove the assumption of a one dimensional, vertical diffusion of CO_2_ inside the amidated pectin solution.

#### 2.1.3. Impact of the CO_2_ Pressure

**Figure 6:**
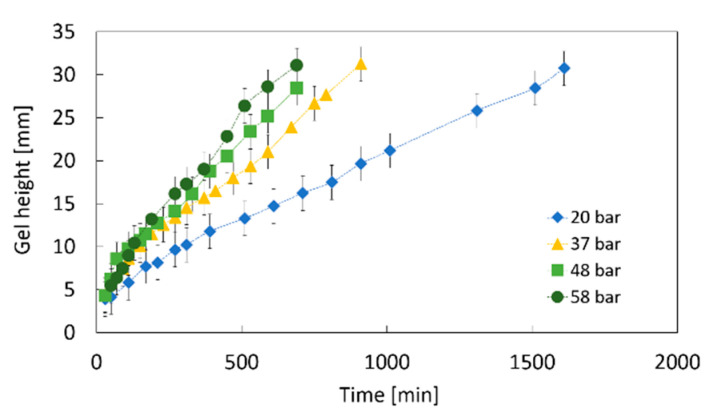
Impact of the CO_2_ pressure in the gas phase on the change in the gel height over time; 1 wt.-% amidated pectin solution with 0.18 wt.-% CaCO_3_ at room temperature.

The influence of the CO_2_ pressure on the gelation process is investigated, since it determines the CO_2_ solubility in the biopolymer solution (step 1, Figure 1). The increase in the gelation height over time is shown in Figure 6 for four different pressures ranging from 20 to 58 bar. The highest gelation rate is observed at the highest pressure. At the same time, for the first ca. 200 min of the process the gelation rate does not vary significantly with pressure. This indicates that at the beginning of the process, the CO_2_ is dissolved rapidly causing fast gelation at the top of the solution. From a certain height onward, the progressively growing gel layer begins to hinder diffusion of CO_2_ into lower parts of the solution. The results shown in Figure 6 indicate that for pressures between 37 and 58 bar the critical height of the gel layer is reached after ca. 200 min. At this point, the diffusion of the CO_2_ is hindered so much that the CO_2_ solubility begins to have an impact on the further gel formation. Thus, at lower pressures, i.e., with lower CO_2_ concentrations at the top of the solution, the CO_2_ diffusion rate decreases as a result of a lower concentration gradient [30].

At 20 bar, a remarkably lower gelation rate is observed directly from the beginning of the process. This indicates that the CO_2_ solubility is so low at this pressure that already a thin gel layer represents a significant hindrance for CO_2_ diffusion. For comparison reasons, the CO_2_ solubility in pure water is shown in Table 1 for studied pressures (values calculated from the Henry’s dissolution constant $H^{CP}=3.3\times 10^{-4}\frac{\mathrm{mol}}{m^{3}\mathrm{Pa}}$ from reference [31] at 25 °C as:

(1) $$\;c=\;H^{CP}\cdot P_{{\mathrm{CO}}_{2}},$$

where c is the concentration of CO_2_ in pure water and $P_{{\mathrm{CO}}_{2}}$ is the partial pressure of CO_2_ in the gas phase.

#### 2.1.4. Impact of the CaCO_3_ Concentration

**Figure 7:**
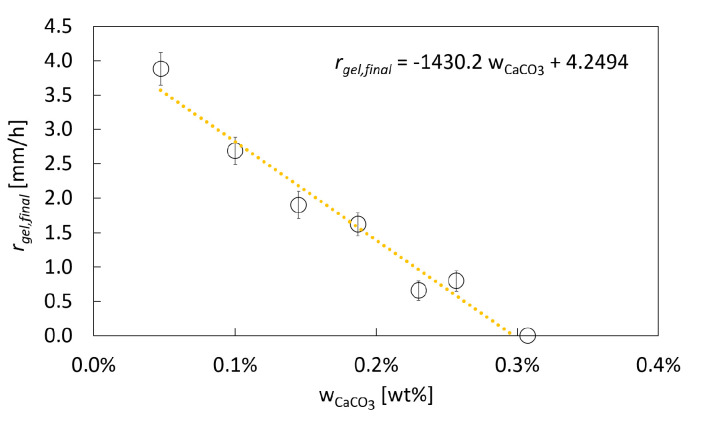
Change in the final gelation rate ($r_{gel,final}$) over initial CaCO_3_ concentration; 1 wt.-% amidated pectin solution, 50 bar, room temperature.

To evaluate the impact of dispersed CaCO_3_, solutions with different CaCO_3_ concentrations are studied with respect to the gel formation and final gelation rate. As shown in Figure 7, the final gelation rate decreases approximately linearly with increasing CaCO_3_ concentration. This can be explained by the fact that the visual observation of the gelation is, strictly speaking, an observation of the dissolution of the CaCO_3_ particles. During the dissolution of CaCO_3_, the pH system is buffered by the reaction of released CO_3_^2−^ ions with the protons to HCO_3_^−^ [19] (Figure 1), decreasing the amount of free protons. Diffusion of more protons towards the gelation front is required for further dissolution of CaCO_3_ and ongoing pH change. At higher CaCO_3_ concentrations, this buffering effect is more pronounced so that it takes longer to dissolve all dispersed CaCO_3_. The approximately linear relation between the initial CaCO_3_ concentration and the final gelation rate (Figure 7) indicates that the dissolution kinetics of single particles are barely influenced, but only the increase in the total amount of CaCO_3_ and therefore the increased need of protons due to stoichiometric reaction causes the decrease in the final gelation rate. For initial CaCO_3_ particle concentrations close to solubility (at process parameters), a lower driving force for dissolution may have an additional impact on the decreased dissolution rate. Hence, a decrease in the final gelation rate occurs with increasing CaCO_3_ concentration up to the solubility of CaCO_3_ in water (0.29 wt.-% at 50 bar CO_2_ and 25 °C; see [22]). If the initial CaCO_3_ concentration is taken above the solubility reached throughout the process, dissolution and gelation process still take place, but cannot be observed anymore due to disturbing residual turbidity of the resulting gels.

With the presented method, no quantification of the amount of dissolved CaCO_3_, besides indication of complete gelation, is possible. In situ quantification of the solution turbidity and the therefore determination of the residual amount of CaCO_3_ is part of our future work to identify the actual cross-linking degree inside the sample at any time and with spatial resolution. Nevertheless, to obtain homogeneous gels, it is recommended to define the cross-linking degree beforehand by the initial CaCO_3_ concentration and perform the gelation process until complete dissolution.

Thus, it could be shown that the kinetics of the cross-linker dissolution have a major impact on the kinetics of the overall gelation mechanism of the biopolymer solution.

The developed method of in situ optical investigation of the gelation process with temporal and spatial resolution enables the detailed investigation of the dissolution and gelation mechanism (step 4 and 5, Figure 1) of the CO_2_-induced gelation. Impact factors such as pressure, composition, and gel formation throughout the gelation process on the kinetics of the overall gelation process can be identified and evaluated.

The optical observation of the gelation system is applicable for all systems with a change in optical appearance, making this method potentially transferable to systems with such a behavior.

### 2.2. In Situ pH Measurement

**Figure 8:**
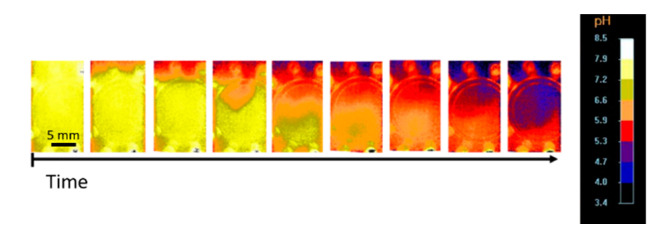
Calculated pH value from fluorescence measurements; 1 wt.-% amidated pectin and 55 bar CO_2_; observable circles and dots at the fluorescence sensor are due to light reflections from LEDs and were not taken into account in the evaluation of pH value.

The pH value of the gelation sample is measured in situ in temporal and spatial resolution inside samples in contact with high-pressure CO_2_ atmosphere using an optical fluorescence system. Pictures of the fluorescence sensor after superimposing the pH value from fluorescence data are shown in Figure 8. At the beginning of the process, a neutral pH value is present in the entire sample as adjusted during solution preparation. The pH value decreases over time from the top of the solution, where it is in contact with gaseous CO_2_, to the bottom, in a good agreement with the one dimensional vertical diffusion of CO_2_ in the sample throughout gelation observation; see Section 2.1.

**Figure 9:**
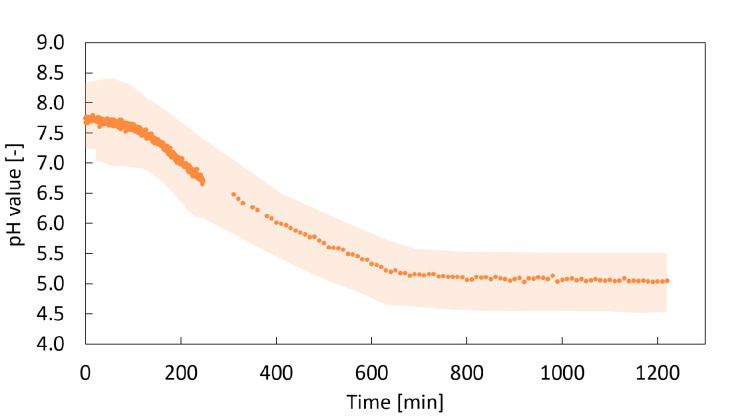
Decrease in the solution pH value over time; 1 wt.-% amidated pectin, 0.18 wt.-% CaCO_3_, 50 bar CO_2_, room temperature; transparent region represents the error margin of pH measurement.

The quantified change in the averaged pH value as a function of time is shown in Figure 9 for a sample of amidated pectin. The pH value decreases from the initial value of around 7.5 to a final, equilibrium pH value of 5.0, indicating dissociation of carbonic acid formed from dissolved CO_2_. The final pH value of 5.0 is sufficient for CaCO_3_ dissolution as it is described in Section 2.1. Although the error margin is comparably high, the trend of the pH value over time can be observed clearly. The equilibrium pH value of 5.0 at 50 bar is in good agreement with literature of the ternary water–CO_2_–CaCO_3_ system [19].

**Figure 10:**
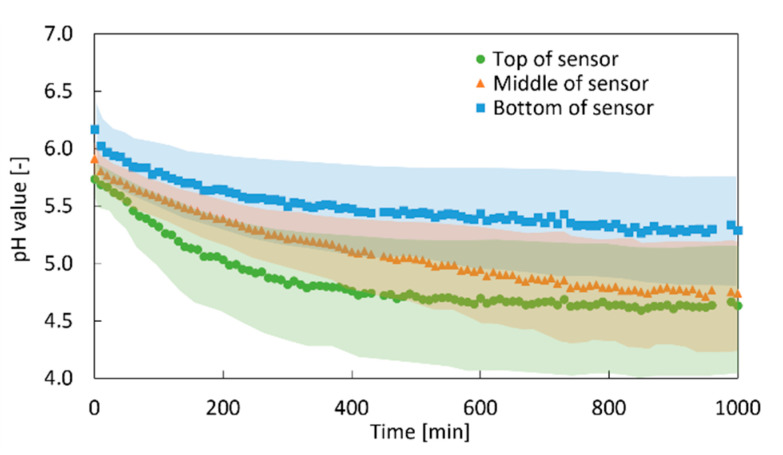
Spatial resolution of the change in the pH value over time; 1 wt.-% amidated pectin solution, 0.18 wt.-% CaCO_3_, 50 bar, room temperature; transparent region represents the error margin of pH measurement.

To determine the change in the pH value with spatial resolution over the sample height, the evaluation of the pH measurements is conducted at different positions of the fluorescence sensor. The results for three regions of the sample (top, middle, bottom) are shown in Figure 10. The pH value starts decreasing at the top of the sensor, followed by the middle part and the bottom part. Although the error margins shown in Figure 10 are large, the trend is clear and consistent with the data obtained from the pH sensor in Figure 8 and the gelation front measurement in Section 2.1. It should be noted that the pH value in the lower regions starts decreasing even before the final pH value is reached at the top of the gel. This indicates that CO_2_ diffuses to lower parts even when CaCO_3_ is not completely dissolved in the upper layers. However, the amount of CO_2_ reaching lower parts is not sufficient to dissolve much of the CaCO_3_ (Section 2.1) until complete gelation of upper parts. At the end of the gelation process, when the sample is completely gelled, a pH value of 5.0 is reached in the entire sample (data not shown).

#### 2.2.1. Impact of the Additional Carbonate

**Figure 11:**
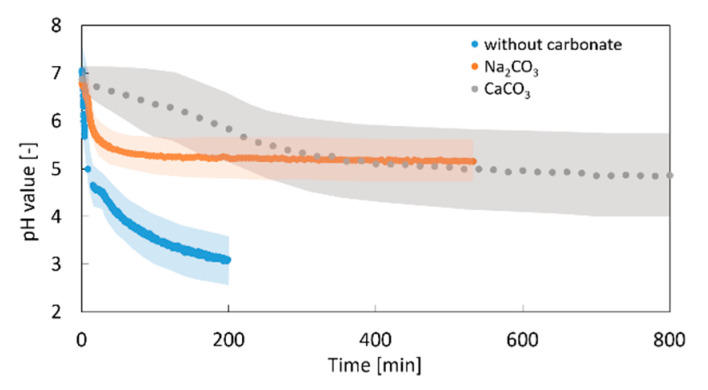
Impact of carbonate salts on the evolution of the pH value under CO_2_ pressure of *p* = 50 bar; 1 wt.-% amidated pectin; 0.18 wt.-% CaCO_3_ or Na_2_CO_3_, respectively; and room temperature. Transparent region represents the error margin of pH measurement.

Two modifications of the experiments are performed to evaluate the impact of CaCO_3_ particles on the evolution of the pH value. First, gelation experiments without CaCO_3_ were conducted, wherein no gelation occurred. Second, substitution of the CaCO_3_ by sodium carbonate (Na_2_CO_3_) is undertaken to study a pure impact of carbonate ions without interference from the gelation and CaCO_3_ dissolution process. The change in the pH value with time in the presence of calcium and sodium carbonates is shown in Figure 11. In this case, the final pH value of the pectin system decreases to approx. 5.0. In contrast, in the system without any additional carbonate ions (excluding the ones formed by the equilibrium of CO_2_ and water), the pH value drops to 3.1. This is in good agreement with the literature values for the binary CO_2_–water reaction systems at 50 bar [32,33]. The result proves that CO_3_^2−^ ions released from dissolution of CaCO_3_ or free CO_3_^2−^ ions from dissociation of Na_2_CO_3_ react with free protons and impact the CO_2_–water reaction equilibrium, demonstrating a buffering effect as shown in the literature for ternary water–CO_2_–CaCO_3_ systems [19,28].

In the case of Na_2_CO_3_, i.e., with free CO_3_^2−^ ions, the pH drop occurs rapidly at the beginning of the process (Figure 11). On the other hand, for dispersed CaCO_3_ a decrease in the pH value is much slower. This might be explained by two different effects: (1) diffusion of CO_2_ in progressively emerging high viscous gel is slowed down (see Section 2.2.2); (2) The CO_2_ diffusion rate is slowed down by the CaCO_3_ dissolution kinetics. Nevertheless, the effect of the CaCO_3_ concentration in the range 0.05–0.23 wt.-% on the pH development kinetics is rather small (data not shown).

#### 2.2.2. Impact of the Solution Viscosity

To investigate the impact of emerging gel layer and concomitant increase in viscosity *η*, three systems with different viscosities are compared. The presence of CO_3_^2−^ ions was realized by addition of Na_2_CO_3_. No gelation takes place due to the absence of Ca^2+^ and thus the viscosity is expected to be constant throughout the process and over the entire sample.

**Figure 12:**
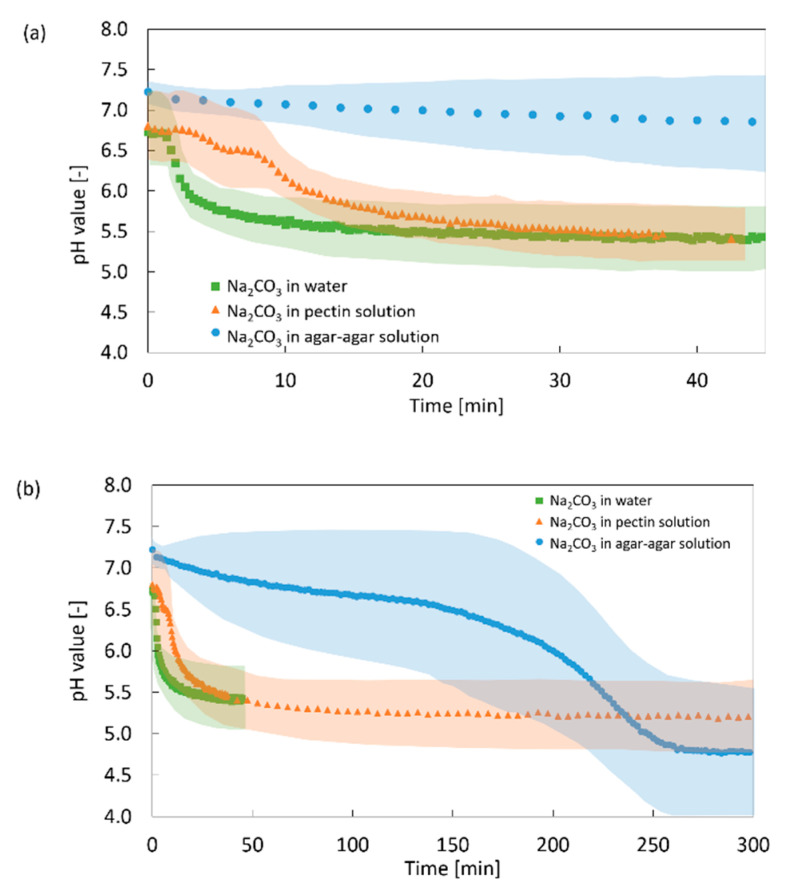
Development of the pH value in aqueous systems with additional Na_2_CO_3_ (0.18 wt.-%) in the presence of CO_2_ (50 bar), water: *η* = 1 mPas, 1 wt.-% amidated pectin: *η* = 30 mPas, 1 wt.-% agar–agar: *η* = 1000 mPas, without CaCO_3_. (**a**) detailed graph of first 45 min; (**b**) complete measurement. Transparent region represents the error margin of pH measurement.

The development of the pH value in water (*η* = 1 mPas), 1 wt.-% amidated pectin solution (*η* = 30 mPas), and 1 wt.-% agar–agar solution (*η* = 1000 mPas) is shown in Figure 12 as a function of time in the presence of CO_2_ at 50 bar. For solutions with higher viscosity, the pH drop is slowed down, indicating the hindrance of CO_2_ diffusion, which is in agreement with general dependence of the diffusion coefficient in the liquid phase on its viscosity according to the Stokes–Einstein equation $D_{AB}\sim \frac{1}{\eta }$. The same impact of the solution viscosity on the pH development is also obtained for systems without additional carbonate sources (data not shown).

In the case of gelling systems, the impact of the hindrance due to increasing viscosity and gel formation is more complex. As discussed in Section 2.1, gelation is much faster than diffusion of CO_2_ (including diffusion of carbonate containing species of HCO_3_^−^ and CO_3_^2−^ and H^+^), resulting in gel formation at the top of the sample and progression of the gelation front to the bottom instead of homogeneous gelation throughout the sample. The formed gel layer hinders diffusion and results in slowed down overall gelation process (compare Figure 3 and Figure 4). Due to progressive gelation for the complex system, the resistance for CO_2_ diffusion is non-homogeneous throughout the sample and changes with time. The complexity of the CO_2_/water reaction system and the lack of information about the location and characteristic time of CO_2_ hydration, acid dissociation (and therefore H^+^ generation), and which species truly diffuse inside the sample complicate the determination of the diffusion rate. Investigation of this more complex case including the quantitative description of CO_2_ diffusion is the matter of our future work.

The in situ measurements of the pH value at high-pressure CO_2_ atmosphere in spatial and temporal resolution enable the investigation of the gelation mechanism step 3 of the overall gelation process (Figure 1) for the amidated pectin model system. The direction and velocity of CO_2_ diffusion and the localization of proton release as well as the impact of the dissolution of the CaCO_3_ are revealed. In combination with the optical observation of CaCO_3_ dissolution, detailed information about the interactions of steps 3 and 4/5 could be obtained. The developed approach is applicable for the investigation of the impact of single components on the development of pH value. Furthermore, the method is transferable to other gelation systems, currently being restricted to the pH range of 2–8, for which sensors are available.

## 3. Conclusions

An experimental set-up combining two different in situ measurement methods was developed to study the mechanism of the CO_2_-induced gelation of biopolymer solutions. The in situ observation of the CaCO_3_ dissolution as well as the monitoring of the pH value of the amidated pectin as a model system were measured in temporal and spatial resolution in CO_2_ atmosphere. Both measurements reveal clearly one-dimensional, vertical diffusion of CO_2_ in the sample from the top (contact area with pressurized CO_2_) to the bottom of the sample. It could be shown that the observed movement of the gelation front is consistent with a measured pH drop throughout the gelation process. The impact of setting parameters, such as pressure, mold material, sample dimensions, and solution composition including cross-linker concentration on the process steps 3 and 4/5, could be identified using the developed in situ methods.

It could be shown that the gel formation itself has a strong impact on the gelation process. The formation of gel layers at the top of the sample caused the hindrance for the CO_2_ diffusion and therefore for the ongoing gelation process. These findings showed that different mechanisms govern the process in different parts of the sample at the same time. For the quantification of the overall gelation kinetics, gelation rates were calculated from the increase in the gel height over time. The gelation rates can be used for the estimation of the gelation duration in samples of different sizes and geometries. The impact of different gelation parameters, such as CO_2_ pressure, solution composition, and geometry and material of the molds on the gelation mechanism, were investigated. It could be shown that the added carbonate directly affects the equilibrium of the CO_2_–water reaction system and therefore the gelation process. Furthermore, it was shown that the kinetics and mechanisms of different process steps depend strongly on the solution composition (CaCO_3_ and CO_2_ content).

Although a few restrictions regarding the change in visual appearance for the optical method and the limited range of the pH measurements reduce the transferability of the presented methods to other biopolymer systems, they can be used for a wide range of reported biopolymer systems to elucidate the impact of single process steps as well as the impact of single components and process parameters on the overall gelation mechanism.

## 4. Materials

Amidated pectin (degree of esterification: 24%; degree of amidation: 25%) was kindly provided by Herbstreith and Fox KG, Neuenbürg/Württ, Germany. Calcium carbonate was kindly provided by Magnesia GmbH, Lüneburg, Germany. Sodium carbonate (Na_2_CO_3_), and agar–agar solutions were purchased from Carl Roth GmbH & Co.KG, Karlsruhe, Germany. Sodium hydroxide (NaOH) was purchased from Th.Geyer, Hamburg, Germany. Sodium chloride (NaCl, food grade) was purchased from Ja! (REWE Markt GmbH), Köln, Germany. CO_2_ was purchased from AGA gas GmbH, Hamburg, Germany. Deionized water was used for solution preparation. All chemicals were used as received.

## 5. Methods

### 5.1. Preparation of Stock Solutions

Amidated pectin (1 wt.-%) was dissolved in deionized water by magnetic stirring and neutralized with 0.5 M sodium hydroxide solution to pH 7. For gelation experiments, calcium carbonate was added to reach a final concentration of 0.05 to 0.23 wt.-% and suspended with a rotor stator homogenizer Heidolph DIAX 900, Heidolph Instruments GmbH & Co.KG, Schwabach, Germany. Agar–agar solution was prepared by dissolution of agar–agar in deionized water by heating up to 95 °C. Biopolymer solutions with 0.18 wt.-% sodium carbonate (Na_2_CO_3_) were prepared. Water–salt mixtures of CaCO_3_ (0.18 wt.-%) and Na_2_CO_3_ (0.2 wt.-%) were prepared by homogenizing the salts in deionized water. Sodium chloride (0.5 to 1.8 wt.-%) was added to reach an appropriate ionic strength for the sensor during pH measurements.

### 5.2. CO_2_-Induced Gelation

**Figure 13:**
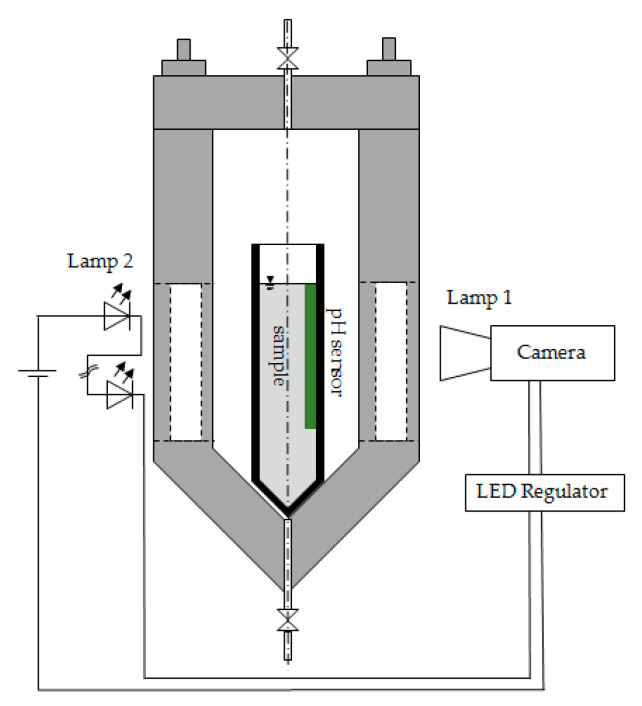
Scheme of the high-pressure viewing cell for CO_2_-induced gelation.

CO_2_-induced gelation was performed in a custom-made high-pressure viewing cell with two vertical opposite windows (Figure 13). Prepared solutions were poured into cylindrical plastic or glass molds with diameters of 1.5, 2.7 or 2.9 cm and placed into the high-pressure viewing cell for gelation or pressurization. Gelation was performed at 10 to 60 bar in CO_2_ atmosphere and at room temperature. The pressure was kept constant for minimum 24 h to guarantee a complete gelation of the sample. Depressurization was conducted afterwards with 1–2 bar/min to avoid bubble formation inside the gel.

### 5.3. Visual Observation and Evaluation of the Gelation Progress

**Figure 14:**
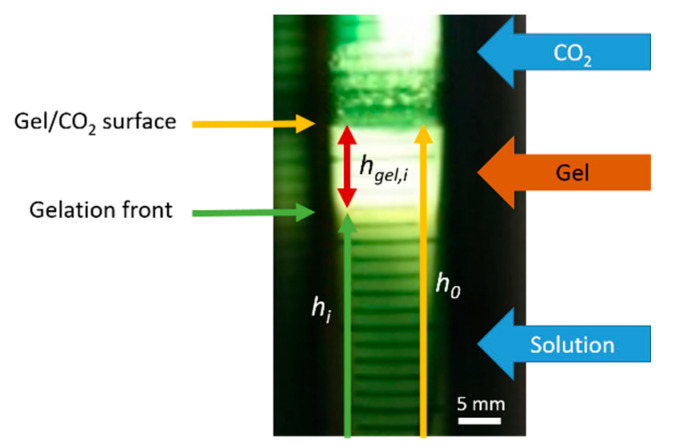
Example for the observation of CO_2_-induced gelation of 1 wt.-% amidated pectin solution inside the viewing cell at 50 bar.

The gelation of the biopolymer solution was observed directly through the windows of the high-pressure viewing cell with back-light from a lamp (lamp 2, Figure 13). Due to the dissolution of the CaCO_3_ particles (step 4, Figure 1), the turbid suspension turned into a clear gel (Figure 14). It could be shown in preliminary experiments that the observed clear regions of the solution consist of stable, completely gelled hydrogel (Figure 14). Therefore, it is assumed that as soon as dissolution of the CaCO_3_ (step 4) can be observed, cross-linking (step 5) already took place.

The progress of the gelation was given by the movement of the gelation front between the turbid solution and the clear gel at the top of the sample (Figure 14). From these observations, the height of already formed gel was calculated as:

(2) $$h_{gel,i}=h_{0}-h_{i}$$

where $h_{gel,i},\;h_{0},\;and\;h_{i}$ are the absolute height of the gel, the position of the gel/CO_2_ phase boundary (measured from the bottom of the sample), and the position of the phase boundary between gel and solution (measured from the bottom of the sample) at time i, respectively (see Figure 14).

The gelation rate describes the time-dependent velocity of the gelation front inside the sample. Two types of gelation rates are distinguished: (1) a total gelation rate $r_{gel,i}$ which is determined as proportion of the formed gel height over a certain time i:

(3) $$r_{gel,i}=\;\frac{h_{gel,i}}{t_{i}-t_{0}},$$

where $t_{i}$ is the time at which the gel height is determined and (2) the gelation rate of a certain period throughout the gelation process determined as the slope of the gel height as a function of time.

(4) $$r_{gel,\;\;period}=\;{\frac{dh_{gel}}{dt}|}_{period}$$

The error of the gel height measurement was assumed as inaccuracy of the reading with ± 2 mm. As common for the evaluation of optical images, measurement values can be biased by the operator’s experience. The error of the calculated gelation rates for time periods was determined as the standard error of the linear regression for linear parts of the plot $h_{gel}$ vs. time.

The development of the gel height and the gelation rate on time is sensitive not only to process parameters such as pressure and temperature, but also to other experimental settings (such as total height of the sample, mold material, and additives such as salts). This should be particularly taken into account when comparing results of obtained values of different measurements.

### 5.4. In Situ pH Measurement

In situ pH measurements were performed with an optical imaging system called VisiSens (PreSens Precision Sensing GmbH, Regensburg, Germany) consisting of fluorescent pH measurement foils, the VisiSens detector unit Duo2 for VisiSens 2, and the evaluation program VisiSens AnalytiCal2 version 2.11. Sticky fluorescent pH foils used as sensors were attached to the internal surface of the sample mold directly in contact with the sample solution. During measurements, the sensor was illuminated by a fluorescent lamp (lamp 1 integrated in the detector unit from the outside of the high-pressure viewing cell, Figure 13). Fluorescence of the sensor is a function of pH value inside the solution. Sensors with pH ranges of 2–4 and 5–8 were used. Spatially resolved fluorescence of the sensor was detected through the window from outside of the autoclave with the camera (Figure 13). Fluorescence was translated to the pH value using a calibration implemented beforehand in the program VisiSens AnalytiCal2.11.

The error margin for the pH measurements at a given time was estimated as the standard deviation across all detected pixels of the fluorescent sensor. To increase accuracy, the viewing cell had to be darkened completely during pH measurements, except for the light from lamp 1 (Figure 13). The pH measurement system enabled the spatial resolution of the pH value over the sample.

### 5.5. Viscosity Measurement

Shear viscosities (*η*) of stock solutions were determined with a Malvern Kinexus Pro (KNX 2100, Malvern Instruments GmbH, Herrenberg, Germany) using a double-slit geometry (DG 25 R0426SS with Df24127) at a shear rate of 1/s.

## Figures and Tables

**Table 1:** Calculated solubility of CO_2_ in pure water at 25 °C; Henry solubility constant of *H^CP^* = 3.3 × 10^−4^ mol/(m^3^ Pa) taken from [31].

Pressure of CO_2_ Atmosphere (Bar)	Solubility of CO_2_ in Pure Water (mol/m^3^)
20	660
37	1221
48	1584
58	1914
